# Readmission Following Revision Total Knee Arthroplasty: An Institutional Cohort

**DOI:** 10.7759/cureus.3640

**Published:** 2018-11-26

**Authors:** Nicholas Dahlgren, Eva Lehtonen, Matthew Anderson, Adam T Archie, Gerald McGwin, Ashish Shah, Sameer M Naranje

**Affiliations:** 1 Orthopaedics, University of Alabama School of Medicine, Birmingham, USA; 2 Orthopaedics, University of Miami, Miller School of Medicine, Miami, USA; 3 Orthopedics, University of Alabama School of Medicine, Birmingham, USA; 4 Epidemiology, University of Alabama School of Medicine, Birmingham, USA

**Keywords:** revision total knee arthroplasty, risk factor, comorbidity, readmission, financial burden

## Abstract

Purpose: Total knee arthroplasty (TKA) is the most common joint replacement surgery performed in the United States. Given the aging and increasingly comorbid patient populations undergoing these procedures, complication rates and the need for subsequent hospital readmission are only expected to rise. It is, therefore, crucial to investigate the risk factors leading to readmission in order to improve patient outcomes. The purpose of this study is to identify significant risk factors for readmission following revision TKA procedures.

Methods: Patients undergoing revision TKA were identified at our institution from 2006-2017. The primary outcome was hospital readmission after revision TKA. Patient demographics, comorbidities, and postoperative complications were recorded and compared between readmitted and non-readmitted patients.

Results: Forty-five (26.2%) of the 171 cases were readmitted following revision TKA. The leading diagnoses at readmission varied from arthrofibrosis in 28.9% of patients, implant infection in 22.2% of patients, and implant failure in 20.0% of patients. Male gender was found to be a significant independent variable for readmission. This study also found that 51.1% of all readmitted patients continued to have complaints that required additional hospital readmissions. The average number of total readmissions was 2.1 per readmitted patient.

Conclusion: This study was successful in identifying variables associated with readmission following revision TKA, as well as presenting information regarding the diagnoses associated with readmission. Our data also showed that if a patient was readmitted after revision TKA, it was likely that they would be admitted again. Due to the increasing prevalence and cost of these procedures, further studies are needed to better understand the risk factors and comorbidities leading to readmission in order to improve the perioperative care of these patients.

## Introduction

Total knee arthroplasty (TKA) is the most common inpatient surgery performed annually in the United States [1]. While generally successful procedures, approximately 8% of patients develop infections, mechanical loosening, or implant failure postoperatively and require revision surgery [2-3]. Given the aging and increasingly comorbid patient populations, the number of revision TKA surgeries performed in the United States is only expected to increase [2,4]. 

Hospital readmissions create a significant financial burden on the health care system, costing an estimated $17.4 billion annually [5]. Readmission following revision TKA has been reported to occur at a rate of 6.2%, which is higher than the readmission rates following primary TKA of 4.6% [6-7]. With the average readmission following TKA estimated to cost over $11,500, it is crucial to investigate the factors leading to readmission in order to improve patient outcomes and to decrease the financial burden placed on health care providers [8].

Recent studies suggest that morbid obesity and major depressive disorder are associated with worse functional outcomes and more complications following revision TKA [9-10]. Additionally, database studies have identified the history of transient ischemic attack or cerebrovascular accident, female sex, and the use of general anesthesia as specific factors contributing to readmission following revision TKA [6]. However, databases are limited to only available data points, and they do not allow for an in-depth look at the events leading to readmission or the outcomes at maximal follow-up. The purpose of this study was to identify the contributing factors, causes, and outcomes of readmission following revision TKA procedures at our institution between 2006 and 2017. We hypothesize that infection and implant failure will be major causes for readmission after revision TKA and hope to identify potential contributing factors to reduce readmissions. 

## Materials and methods

This is a retrospective study that was approved by the Institutional Review Board in accordance with the Declaration of Helsinki. The medical records of a randomized sample of patients undergoing revision TKA at our institution between January 2006 and December 2017 were screened for inclusion in this study. Patients with single and multiple staged TKA revisions were included, while those with primary TKA, amputation, and oncology-based reconstructions were excluded. 

The medical records were then reviewed and data were extracted for patients who met the study inclusion criteria. The primary outcome of interest was hospital readmissions following revision TKA. Other extracted data included patient age, sex, race, body mass index (BMI), the presence of comorbid medical conditions at the time of surgery, American Society of Anesthesiologists (ASA) classification, length of hospital stay, and postoperative medical and surgical complications. Comorbid medical conditions of interest included obesity, diabetes mellitus, hypertension, coronary artery disease (CAD), congestive heart failure (CHF), chronic kidney disease (CKD), chronic obstructive pulmonary disease (COPD), deficiency anemia, peripheral vascular disease (PVD), liver disease, human immunodeficiency virus (HIV) status, thyroid disease, major depressive disorder (MDD), and cerebrovascular disease. Postoperative complications included medical complications such as pneumonia, unplanned intubation, urinary tract infection (UTI), ventilator use for greater than or equal to 48 hours, pulmonary embolism, acute renal failure, cardiac arrest, myocardial infarction, stroke or cerebrovascular accident (CVA), blood transfusion, and surgical complications including superficial incisional surgical site infection (SSI), deep incisional SSI, organ space SSI, and wound disruption. 

Patient demographics, preoperative comorbidities, and postoperative medical and surgical complications were compared between readmitted and non-readmitted patients using Wilcoxon–Mann–Whitney and Kruskal Wallis tests for continuous variables and Fisher's Exact test for categorical variables, respectively. A p-value of 0.05 was used for statistical significance. 

## Results

Patient characteristics

A total of 171 patients were included in this study. Reasons for revision TKA included implant failure in 120 (70.2%) patients, infection in 47 (27.5%) patients, periprosthetic fracture in three (1.8%) patients, and painful primary TKA in one (0.6%) patient. The majority (76.6%) of patients had no history of previous revision TKA, while 34 (19.9%) patients had a history of one previous revision, four (2.3%) patients had a history of two previous revisions, one (0.6%) patient had a history of three previous revisions, and one (0.6%) patient had a history of seven previous revision surgeries. The characteristics of included patients are shown in Table 1. 

**Table 1:** Preoperative characteristics of readmitted and non-readmitted total knee arthroplasty patients Percentages indicate percent of patients within each readmission status group (non-readmitted or readmitted). BMI = body mass index; CHF = congestive heart failure; COPD = chronic obstructive pulmonary disease; WBC = white blood cells; INR = International Normalized Ratio; ASA = American Society of Anesthesiologists

	Non-Readmitted	Readmitted	P-Value
Number of Patients (N=171)	126	45	
Demographic Characteristics			
Mean age (in years (range))	63.42 (37.4-88.44)	62.53 (34.15-79.52)	0.9176
Sex (in number of patients (%))			0.0131
Male	46 (36.51%)	26 (57.78%)	
Female	80 (63.49%)	19 (42.22%)	
Race (in number of patients (%))			0.5781
African American	42 (33.33%)	12 (26.67%)	
White	83 (65.87%)	33 (73.33%)	
Not Reported	1 (0.79%)	0 (0.00%)	
BMI Category (in number of patients (%))			0.5658
Underweight (<18.5 kg/m^2^)	2 (1.59%)	0 (0.00%)	
Normal (18.5 to <25 kg/m^2^)	12 (9.52%)	5 (11.11%)	
Overweight (25 to <30 kg/m^2^)	39 (30.95%)	10 (22.22%)	
Obese (30 to <35 kg/m^2^)	29 (23.02%)	16 (35.56%)	
Very obese (35 to < 40 kg/m^2^)	24 (19.05%)	7 (15.56%)	
Morbidly Obese (≥ 40 kg/m^2^)	20 (15.87%)	7 (15.56%)	
Preoperative comorbidities (in number of patients (%))			
Recent Weight Loss	0 (0.00%)	0 (0.00%)	
Current Smoker	14 (11.11%)	9 (20.00%)	0.1336
Diabetes			0.5887
Diabetes: Insulin-Dependent	6 (4.76%)	4 (8.89%)	
Diabetes: Non-Insulin	19 (15.08%)	7 (15.56%)	
Dialysis	0 (0.00%)	1 (2.22%)	0.0933
Hypertension	85 (67.46%)	29 (64.44%)	0.7126
Dyspnea on Exertion	15 (11.90%)	3 (6.67%)	0.3257
CHF	0 (0.00%)	0 (0.00%)	
COPD	7 (5.56%)	1 (2.22%)	0.3634
Bleeding Disorders	5 (3.97%)	1 (2.22%)	0.5848
Corticosteroid Use	5 (3.97%)	3 (6.67%)	0.4619
Preoperative Lab Results (mean measured value (range))			
WBC (cells/mm^3^)	7.00 (3.2-14.9)	8.13 (2.50-24.0)	0.0677
Hematocrit (%)	39.45 (24.0-53.0)	38.93 (27.0-48.0)	0.6241
Platelet Count (cells x 10^9^/L)	247.90 (120.0-463.0)	279.91 (85.0-749.0)	0.2188
Serum Creatinine (mg/dL)	0.94 (0.40-1.70)	1.13 (0.50-6.80)	0.4679
Albumin (g/dL)	3.93 (2.90-4.50)	3.93 (3.40-4.60)	0.818
INR	1.27	1.11	0.6118
ASA class (in number of patients (%))			0.122
ASA 1	0 (0.00%)	1 (2.22%)	
ASA 2	23 (18.25%)	6 (13.33%)	
ASA 3	102 (80.95%)	36 (80.00%)	
ASA 4	1 (0.79%)	2 (4.44%)	
Number of previous revisions			
1	23 (18.25%)	11 (24.44%)	
2	3 (2.38%)	1 (2.22%)	
3	1 (0.79%)	0 (0.00%)	
7	0 (0.00%)	1 (2.22%)	

Postoperative outcomes

Mean duration of clinical follow-up for all patients was 18.6 months (range, 0.5-131.4 months). Six patients were lost to follow-up after the revision procedure. Four (2.3%) patients were readmitted within 30 days of revision TKA and 45 (26.3%) patients were readmitted within 90 days. 

Postoperatively, there was one case of superficial incisional surgical site infection (SSI), two cases of deep incisional SSI, and two cases of deep organ space SSI. Medical complications included one case of postoperative pneumonia, one case of postoperative UTI, one case of myocardial infarction, and one case of venous thrombosis. Twenty-four patients required blood transfusions intraoperatively or within 72 hours of operation. Of these, 12 patients required one unit, six patients required two units, one patient required three units, three patients required four units, and one patient required five units. There was one case of sepsis. No other complications were reported, including no unplanned intubations, no pulmonary embolism, no ventilator use greater than 48 hours, no *Clostridium difficile *(C. diff) colitis, no progressive renal insufficiency or acute renal failure, no stroke or CVA, no cardiac arrest, and no death following revision TKA. Outcomes of readmitted and non-readmitted patients are shown in Table 2. 

**Table 2:** Postoperative outcomes of readmitted and non-readmitted total knee arthroplasty patients Percentages indicate percent of patients within each group (non-readmitted or readmitted). UTI = urinary tract infection

	Non-readmitted	Readmitted	p-value
Hospital length of stay (in days, mean (range))	3.87 (2.00 - 27.00)	3.96 (2.00 - 10.00)	0.8509
Medical complications (in number of patients (%))			
Pneumonia (%)	0 (0.00%)	1 (2.22%)	0.0933
UTI (%)	1 (0.79%)	0 (0.00%)	0.5489
Myocardial Infarction (%)	1 (0.79%)	0 (0.00%)	0.5489
Blood Transfusion within 72 hours of surgery (%)	17 (13.49%)	7 (15.56%)	0.7323
Surgical complications (in number of patients (%))			
Superficial Surgical Site Infection	0 (0.00%)	1 (2.22%)	0.0933
Deep Surgical Site Infection	0 (0.00%)	2 (4.44%)	0.0681
Organ or space surgical site infection	1 (0.79%)	1 (2.22%)	0.4442

Readmitted patients

**Figure 1:**
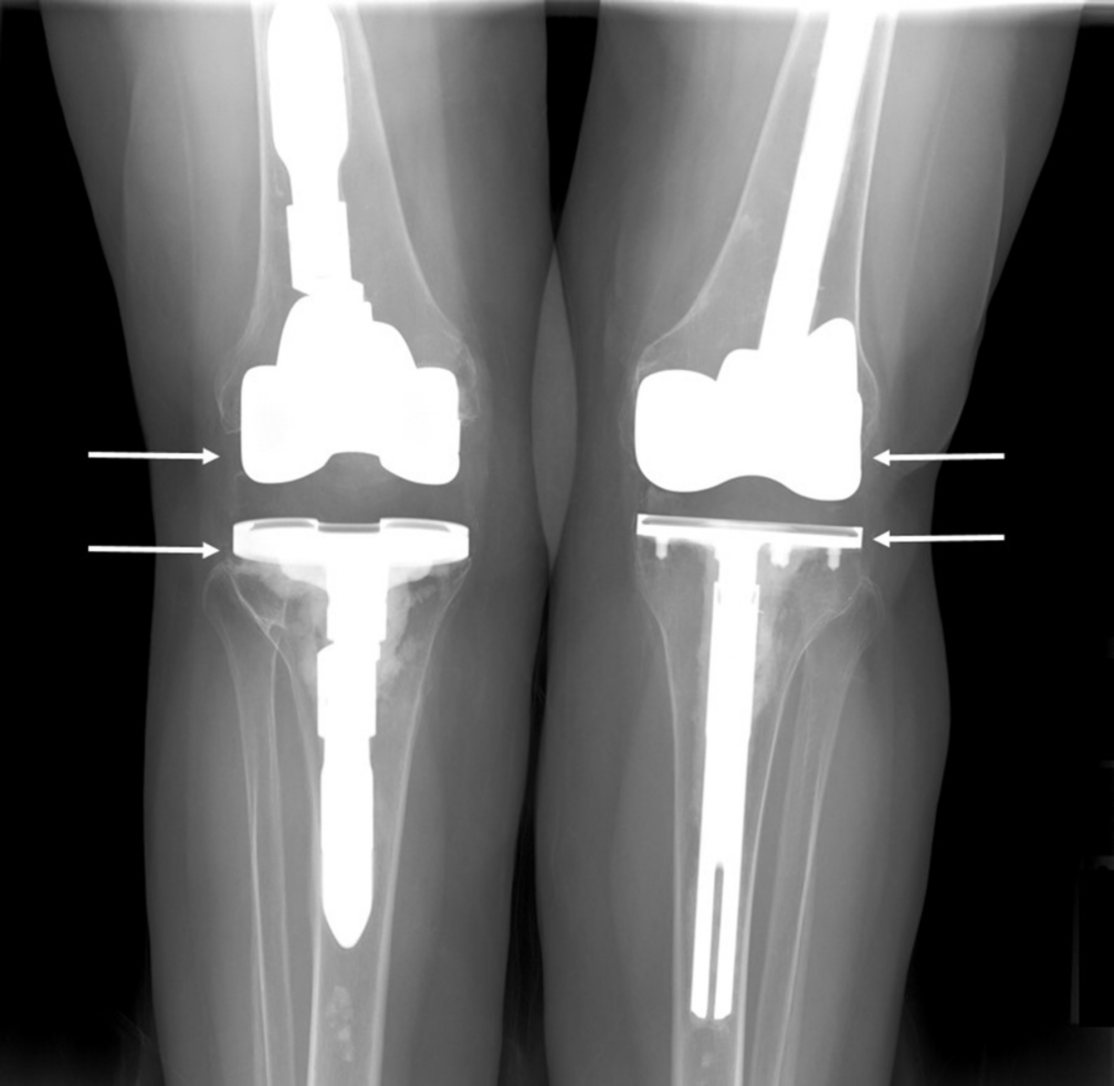
Preoperative X-ray showing hardware prior to bilateral revision total knee arthroplasty for instability (instability not visible on imaging)

**Figure 2:**
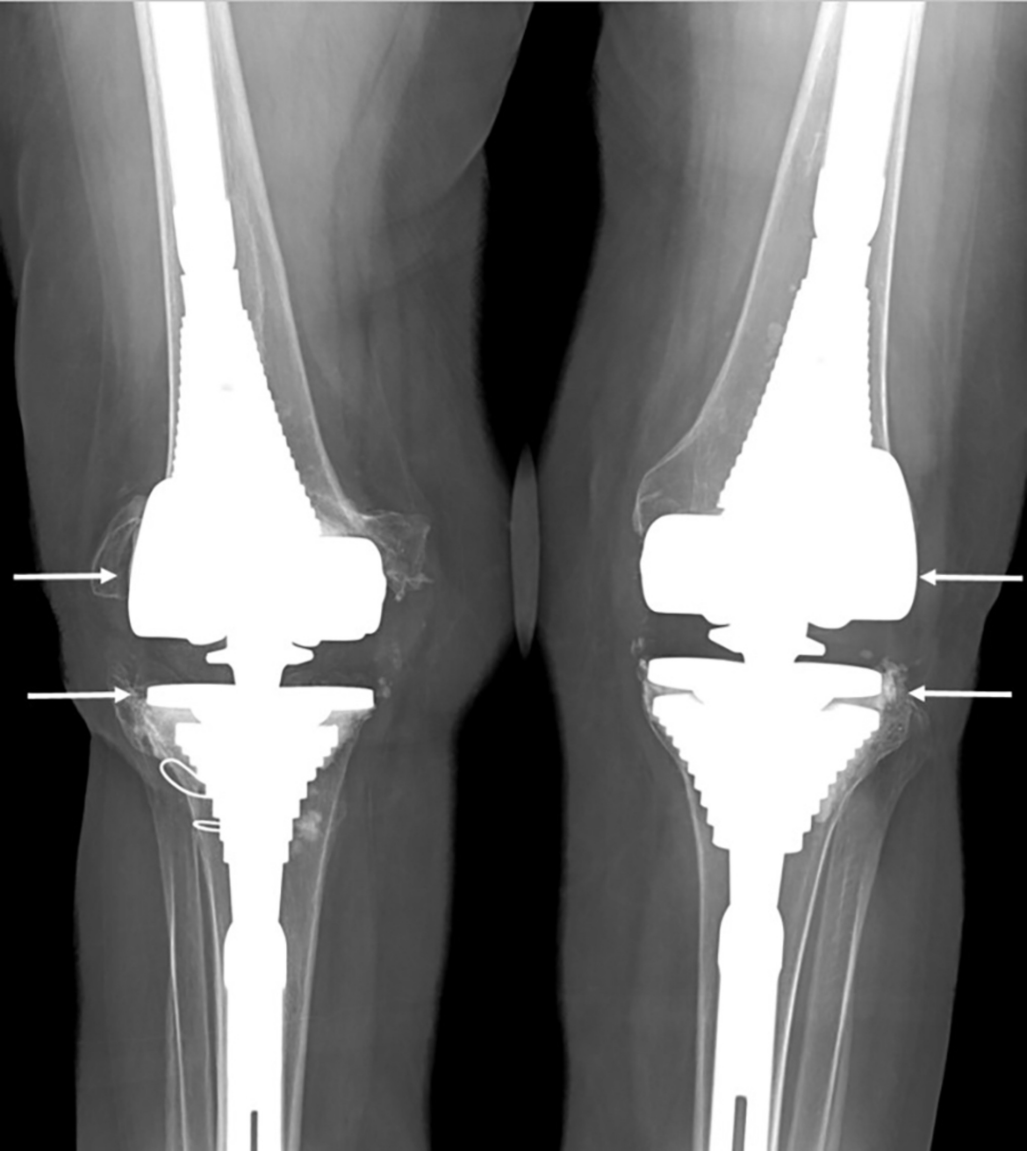
Postoperative X-ray showing intact hardware following bilateral revision total knee arthroplasty for instability

Forty-five (26 male, 19 female) patients were readmitted after revision TKA. Reasons for readmission varied from arthrofibrosis in 13 (28.9%) patients, implant infection in 10 (22.2%) patients, implant failure in nine (20.0%) patients, instability in five (11.1%) patients, wound abscess or dehiscence in four (8.9%) patients, periprosthetic fracture in one (2.2%) patient, retained foreign body in one (2.2%) patient, popliteal mass in one (2.2%) patient, and post-operative hematoma in one (2.2%) patient. The outcomes and success of these readmissions also varied, as 23 (51.1%) of the readmitted patients continued to have complaints that ultimately required one or more additional hospital readmissions to address. The average number of total readmissions was 2.1 per readmitted patient (range: 1-6). Despite these setbacks, 32 (71.1%) patients were documented as satisfied or doing well at their final clinic follow-up visit. Pre-operative and post-operative X-rays demonstrating successful revision TKA are shown in Figures 1-2. 

## Discussion

The volume of TKAs performed annually is steadily increasing. Given this trend and the increasing number of patients with multiple comorbidities undergoing these procedures, complication rates and the need for subsequent revision surgeries and hospital readmission are also expected to rise [2-3]. Properly identifying and addressing comorbidities that predispose to poor functional outcomes may allow for better perioperative care and reduction of readmission rates in patients scheduled to undergo revision TKA. Understanding and mitigating these risk factors may provide public health benefits not only to patients but also to surgeons, hospitals, and health care as a whole [2,8]. 

Our study found that male gender was a significant predictor of hospital readmission following revision TKA. This finding is consistent with a similar study performed by Cram et al., which also showed a significant association between male gender and increased risk of readmission after revision TKA [4]. In another publication, Badawy et al. found that male patients undergoing primary TKA had two times relative risk of revision due to deep surgical site infection compared to female patients [11]. It is possible that the size of our study prevented us from detecting the previous finding, as we were unable to perform multivariate analysis of our data.

There is still limited literature pertaining to risks for readmissions following revision TKA. Belmont et al. found that transient ischemic attack/cerebrovascular accidents (TIA/CVAs) and the use of general anesthesia were predictors of hospital readmission following TKA [6]. They concluded that surgeons should consider neuraxial anesthesia over general anesthesia and should facilitate a proper transition of care in patients with a history of TIA/CVA [6]. Given that no patients in our cohort had a past medical history of cerebrovascular disease and that our study did not include anesthesia modalities, we were not able to appreciate these findings.

It is widely accepted that obesity and BMI can influence surgical outcomes [1,9,12]. Watts et al. found that patients with morbid obesity (BMI>40 kg/m^2^) had a higher prevalence of additional complications and subsequent TKA revisions when compared to matched patients with BMI less than 30 kg/m^2^ [9]. Another study by Kremers et al. demonstrated the financial impact associated with obesity and readmissions after revision TKA [13]. They reported a higher average hospital cost of $645 per 5-unit increase in BMI>30 kg/m^2^. Excess costs associated with BMI were higher for revision TKA than those calculated for primary TKA, highlighting the importance of mitigating the need for subsequent readmissions following revision TKA. It is worth noting that the majority (66.7%) of patients readmitted in our study had a BMI>30 kg/m^2^, however, this variable failed to reach statistical significance [13]. 

This study does not report any additional variables that are significantly associated with readmission following revision TKA. However, our data did show that if a patient was readmitted after revision TKA, it was likely that they would be admitted again. Among readmitted patients, the average number of readmissions was 2.1 (range 1-6); of 45 readmitted patients, 23 (51.1%) patients were readmitted more than once. Our data showed that a majority of patients who were readmitted multiple times had an initial readmission diagnosis of implant failure or complication due to arthrofibrosis (eight of 23, 34.8%), implant infection (six of 23, 26.1%), or implant failure due to other unspecified cause (three of 23, 13.0%).

The results of this study were composed under certain limitations. The study was performed using data from a single academic institution in the Southeastern United States with non-diverse population demographics. The majority of included patients were Caucasian (67.8%) or African-American (31.6%). Additionally, the number of patients meeting the inclusion criteria at our institution limited the power of this study and restricted the data to univariate analyses. This study did not include the entire population at our institution; we selected a randomized sample to make detailed chart review practical. A larger sample size would have allowed for a more accurate scope and detection of statistically significant variables associated with readmission after revision TKA.

## Conclusions

This study was successful in identifying variables associated with readmission following revision TKA, as well as presenting new information regarding the diagnoses associated with multiple readmissions. Physicians can use this data clinically to recognize patient populations at risk of poor outcomes following revision TKA. Due to the increasing prevalence and cost of these procedures, it is critical to minimize the number of additional hospital readmissions and the need of subsequent revisions. Further studies should be directed at better understanding these risk factors and comorbidities in order to improve patients' perioperative care and quality of life.
